# Inference of Cross-Level Interaction between Genes and Contextual Factors in a Matched Case-Control Metabolic Syndrome Study: A Bayesian Approach

**DOI:** 10.1371/journal.pone.0056693

**Published:** 2013-02-20

**Authors:** Shi-Heng Wang, Wei J. Chen, Lee-Ming Chuang, Po-Chang Hsiao, Pi-Hua Liu, Chuhsing K. Hsiao

**Affiliations:** 1 Institute of Epidemiology and Preventive Medicine, College of Public Health, National Taiwan University, Taipei, Taiwan; 2 Genetic Epidemiology Core Laboratory, Center of Genomic Medicine, National Taiwan University, Taipei, Taiwan; 3 Department of Public Health, College of Public Health, National Taiwan University, Taipei, Taiwan; 4 Department of Internal Medicine, National Taiwan University Hospital, Taipei, Taiwan; 5 Institute of Clinical Medicine, College of Medicine, National Taiwan University, Taipei, Taiwan; 6 Clinical Informatics and Medical Statistics Research Center, Chang Gung University, Guishan, Taiwan; 7 Bioinformatics and Biostatistics Core, Center of Genomic Medicine, National Taiwan University, Taipei, Taiwan; Tor Vergata University of Rome, Italy

## Abstract

Genes, environment, and the interaction between them are each known to play an important role in the risk for developing complex diseases such as metabolic syndrome. For environmental factors, most studies focused on the measurements observed at the individual level, and therefore can only consider the gene-environment interaction at the same individual scale. Indeed the group-level (called contextual) environmental variables, such as community factors and the degree of local area development, may modify the genetic effect as well. To examine such *cross-level interaction* between genes and contextual factors, a flexible statistical model quantifying the variability of the genetic effects across different categories of the contextual variable is in need. With a Bayesian generalized linear mixed-effects model with an unconditional likelihood, we investigate whether the individual genetic effect is modified by the group-level residential environment factor in a matched case-control metabolic syndrome study. Such cross-level interaction is evaluated by examining the heterogeneity in allelic effects under various contextual categories, based on posterior samples from Markov chain Monte Carlo methods. The Bayesian analysis indicates that the effect of rs1801282 on metabolic syndrome development is modified by the contextual environmental factor. That is, even among individuals with the same genetic component of *PPARG*_Pro12Ala, living in a residential area with low availability of exercise facilities may result in higher risk. The modification of the group-level environment factors on the individual genetic attributes can be essential, and this Bayesian model is able to provide a quantitative assessment for such cross-level interaction. The Bayesian inference based on the full likelihood is flexible with any phenotype, and easy to implement computationally. This model has a wide applicability and may help unravel the complexity in development of complex diseases.

## Introduction

Both genetic and environmental factors play an important role in the development of many diseases, often interacting in ways that elevate disease risk, especially in complex diseases such as obesity, cardiovascular disease, and psychopathology [1]–[3]. To investigate genetic and environmental (GE) interactions [4]–[6], many studies have focused on environmental factors measured at the individual level, such as individual demographic information or lifestyle. It is possible, however, that group-level environmental factors, usually called contextual variables, also contribute to such risk. For instance, community disparity in medical resources may affect the availability and quality of treatment, and therefore living in a deprived area may result in a somewhat modified effect on the health risk incurred by individuals who engage in unhealthy behaviors such as smoking [7]. This contextual exposure affects the individual health status of each member of the group, and is therefore called the *contextual effect* [8], [9].

Difficulties arise when it is of interest to model the *cross-level interaction* between the group and individual variables, especially with data of nested structures. Traditional logistic regression models with health status as the unit of analysis can include both contextual variables and genes as the covariates, but this approach is not appropriate for nested data where individuals within the same group or under the same category may share a certain degree of similarity in risk profile [8], not to mention when this similarity is group- or category-specific. Even conditional logistic regression models are not flexible enough to account for the nested structure of heterogeneity in such a study design. Other issues concern the evaluation and computation of the cross-level interaction. These issues differ from what has been considered in most current research where GE interaction is evaluated at the individual level based on a case-control study design. For cross-level GE interaction in clustered data, one immediate computational complexity involves the existence of multiple sources of variation. For example, homogeneity among individuals in the same group may produce dependence [8], whereas the corresponding matched controls for any case may induce further correlation. In addition, the genetic effects may be modified by group-level exposures and may, therefore, correlate with each other. These nested sources of variability hamper the model construction and parameter inference. Several research groups adopted Bayesian or non-Bayesian hierarchical models to alleviate the problem of nested variability [10], [11], but these models are not for matched case-control studies and not for group-level GE interaction [12]–[15].

To accommodate the nested structure of variation in both the individual- and group-level variables, and to model directly the interaction between individual genetic factors and group-specific variables, Bayesian hierarchical models with random effects can be formulated for inference. By employing hierarchical models with mixed effects, inference of these multiple variance components becomes feasible and may help elucidate whether cross-level interaction does exist, and/or quantify the strength of this interaction from a probabilistic perspective based on posterior distributions.

This research was motivated by a matched case-control study of metabolic syndrome [16], a condition which is characterized by a group of metabolic conditions and risk factors. In that metabolic disorder study, the effects of community exercise facility as well as five candidate SNPs (rs1801282, rs7799039, rs12535708, rs822390, and rs182052) were investigated. These factors were selected based on previous publications of association studies linking them to obesity and body mass index (BMI). Many studies have recognized the association between metabolic disorder and both diabetes and coronary heart disease [17], [18], while a few studies have shown that both genes and contextual variables, such as community disadvantage, income inequality, and factors affecting community development [19]–[22], associate strongly with the risk of metabolic syndrome. In a densely populated country such as Taiwan where the boundary between residential areas and commercial districts is blurred, an individual household may not have enough space for exercise. Even at the community level, there may not be sufficient exercise facilities such as gymnasiums, track fields, parks, or playgrounds for residents to use. This may restrict an individual’s accessibility to an exercise facility and influence one’s degree of physical inactivity. Although such group-level environmental variables can exert an effect on the risk of metabolic syndrome in addition to the effect exerted by individual environmental factors [19]–[23], it is not certain whether the contextual variable will interact with the genetic effect in the same manner as the individual environmental variable does [24]–[29]. In the rest of this article, we reserve the abbreviation GE for the cross-level interaction at the contextual-level, and Ge for the gene-environment interaction at the individual-level.

The purpose of this paper is to explore the cross-level GE interaction in this matched case-control study via a Bayesian hierarchical model with an unconditional likelihood approach. We illustrate our approach with the aforementioned study concerning five candidate SNPs and GE which involves a contextual variable measuring the availability of exercise facilities. The posterior samples of the parameters of interest, the group-specific random effects and the variance among them, will be used to examine the existence of cross-level GE interaction.

## Methods

### Motivating Example: The Metabolic Syndrome Study

This study was conducted by Wang and colleagues in Tao-Yuan County in Taiwan in 2004 [16]. Here we briefly summarize the collection procedures of samples, while other details are referred to their original paper. This study recruited 6463 community residents aged greater than 40 years old in an adult health check-up program, and collected a questionnaire and blood sample from each participant. Among the recruited subjects, 1263 were classified with metabolic syndrome based on US National Cholesterol Education Program Adult Treatment Panel III (NCEP ATP III) criteria, with BMI replacing waist measurements. That is, an individual was considered to have metabolic disorder if three or more of the following criteria were met: BMI ≥ 27 kg/m^2^; triglycerides ≥ 150 mg/dl; HDL <40 mg/dL in men and <50 mg/dL in women; blood pressure ≥ 130/85 mmHg or medication for hypertension currently taken; and fasting glucose ≥ 110 mg/dL or medication for diabetes currently taken. Next, two hundred- and sixty-eight cases were randomly selected and matched with one or two controls by sex, age, educational level, ethnicity, and a contextual variable measuring the availability of exercise facilities. Exercise facilities included swimming pools, parks, bowling alleys, golf courses, fitness centers, and activity centers for the elderly. For each residential area, the exercise facility density was defined as the number of facilities divided by the number of residents, and this density was categorized into four different levels of availability in exercise facility: level I for very low availability, II for low, III for medium, and IV for high. The final data set for analysis contained 268 metabolic syndrome cases and 322 individually matched controls. The terms “cases” and “controls” here do not intend to indicate that metabolic syndrome is a disease. It is indeed a group of metabolic conditions that associate strongly with increased risk of cardiovascular disease and diabetes. In other words, *metabolic syndrome* is a clustering of risk factors. Discussions and debates about its definition, inclusion criteria, and usefulness in clinical practice have drawn much attention [30], [31]. Here we call these identified individuals “cases” simply for ease of presentation.

### Candidate Genes and Odds Ratios

Three candidate genes, the adiponectin gene *APM1* on chromosome 3q27, the peroxisome proliferator-activated receptor γ gene *PPARG* on 3p25, and the leptin gene *LEP* on 7q31, were selected as candidate susceptibility genes for human obesity and Type 2 diabetes [32]–[34], and five SNPs (rs1801282 on *PPARG*, rs7799039 and rs12535708 on *LEP*, and rs822390 and rs182052 on *APM1*, respectively) were genotyped for this study. Based on preliminary analysis of genotype-specific odds ratios, we employed an additive model for each of the three SNPs, rs1801282, rs182052 and rs822390, and a recessive model for the other two. Table 1 lists the distributions of the SNP genotypes as well as their genetic effects in terms of conditional odds ratios of GE interaction under each category of the contextual variable. It can be observed that the category-specific genetic effect seems to vary across the four levels, indicating a possible GE interaction. Furthermore, because linkage disequilibrium (LD) was observed between rs12535708 and rs7799039 (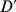 = 0.89, R-square = 0.46), the SNP-SNP interaction between them is considered in later analysis. In addition, because a protective effect in carriers of the Ala allele of rs1801282 (*PPARG*_Pro12Ala) against Type 2 diabetes and its association with lower body mass [35], [36] has not been consistently reported [37], possible environmental modifiers like exercise may be considered [38]. Therefore, in the following we focus on the cross-level interaction between rs1801282 and exercise facility availability in the residential environment.

**Table 1:** The observed genotype counts of metabolic cases (cs) and controls (cn) under each category of exercise facility availability.

	Category				Total
SNP	I	II	III	IV	
genotypes (coding)	cs/cn	cs/cn	cs/cn	cs/cnl	cs/cn
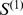 , rs1801282, *PPARG*_Pro12Ala					
C/C (0)	46/52	84/109	63/68	51/64	244/293
C/G (1)	5/1	10/13	4/8	5/7	24/29
Conditional OR	5.7	0.9	0.7	0.9	
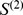 , rs7799039, *LEP*_G2548A					
A/A, A/G (0)	40/45	77/108	54/69	42/62	213/281
G/G (1)	11/8	17/17	13/7	14/9	55/41
Conditional OR	1.4	1.4	2.7	3.3	
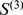 , rs12535708, *LEP*_H1328082					
C/C, C/A (0)	50/50	83/114	60/73	52/68	245/305
A/A (1)	1/3	11/8	7/3	3/4	23/17
Conditional OR	0.4	2.3	2.9	1.8	
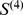 , rs822390, *APM1*_G_7950T					
T/T (0)	41/47	83/114	58/72	45/65	227/298
T/G (1)	6/5	6/4	6/4	2/3	20/16
G/G (2)	4/1	5/4	3/0	9/3	21/8
Conditional OR	1.7	1.6	3.2	2.0	
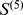 , rs182052, *APM1*_A_10066G					
A/A (0)	6/8	13/26	7/18	11/15	37/67
A/G (1)	28/23	46/62	30/38	25/38	129/161
G/G (2)	17/22	35/34	30/20	20/18	102/94
Conditional OR	0.9	1.5	1.8	1.3	
Total	51/53	94/122	67/76	56/71	268/322

### Bayesian Hierarchical Model with Unconditional Likelihood

To examine if the effect of the SNP rs1801282 varies among different categories of the contextual covariate, along with the SNP-SNP interaction between rs12535708 and rs7799039 in the same gene *LEP* on 7q31, we adopt a Bayesian generalized linear mixed-effects model (GLMM). This Bayesian GLMM assumes that each response variable 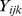, the binary disease status (1 for case and 0 for control) of the 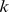-th individual in the 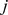-th pair of the 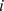-th category, follows a Bernoulli distribution with the parameter 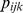 indicating the probability of having metabolic syndrome,
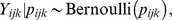
where the probability of being diseased 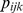 is associated in the logit scale with the 5 (SNP) genetic components 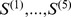 (following the same order and coding as in Table 1) and a SNP–SNP interaction 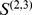, in addition to other covariates 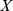,



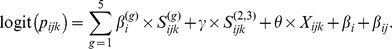



The implication of each parameter is explained in the following paragraphs, and outlined in the supporting information Table S1. All considered explanatory variables are summarized as well.

### Cross-level GE Interaction

The covariate 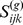 indicates the coding of the SNP 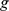 for the 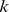-th individual in the 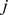-th pair of the 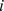-th category. Its associating parameter 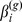 represents the SNP effect under the 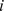-th category for the SNP 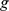. For instance, the evaluation and comparison of the 4 (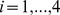) posterior distributions of 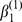, 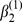, 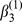, and 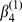 can reveal if the effect of the first SNP (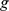 = 1) rs1801282 varies among the 4 different categories of the contextual covariate. Therefore, the variance var(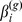) = 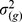 can model directly the heterogeneity in SNP effects among 4 categories for each SNP 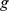. This variance measures the degree to which the genetic effect differs across different categories of the contextual variable. This is thus the parameter of interest when the cross-level GE interaction is to be evaluated.

### SNP-SNP Interaction and Other Effects

The SNP-SNP interaction between the second SNP rs7799039 and the third SNP rs12535708 in the same gene is measured by 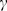. The parameter 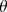 represents the effect of other explanatory variable 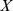. The random intercept 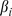 stands for the category-specific effect accounting for the sampling variability among residential areas. The second random intercept 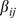 is for the pair- or cluster-specific random effect. This 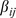 represents the matching relation within each pair. In other words, subjects in the same pair share the same baseline characteristics and pairs are taken to be independent. The random coefficients are all assumed to follow normal distributions with a large variance representing vague information. A complete model specification including all prior distributions is detailed in the supporting information (Text S1).

### Computation

The analysis was conducted based on posterior samples obtained from Markov chain Monte Carlo (MCMC) methods with WinBUGS 1.4.3. The chain contained 50,000 iterations following a burn-in of 5,000 samples to reduce the impact from initial values and the final posterior sample was derived at the thinning rate of 10 to reduce dependence among posterior points. The code in WinBUGS is detailed in the supporting information (Text S2).

## Results

### Cross-level Gene-Environment Interaction

**Figure 1:**
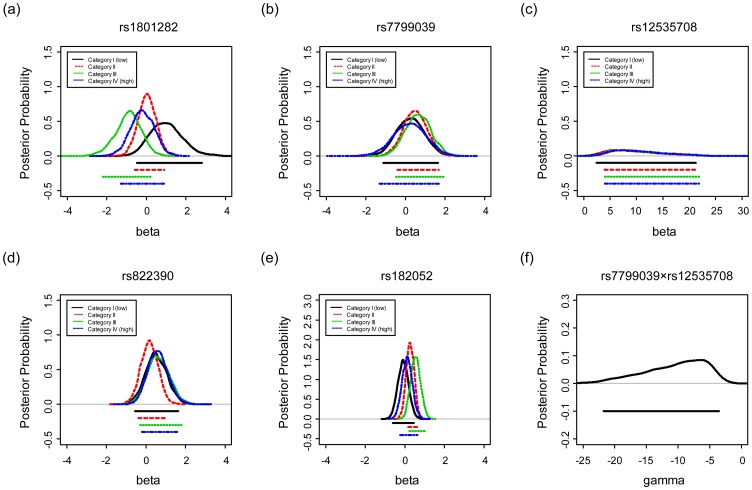
The posterior distributions of 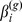 (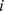 = 1,…,4) for four categories are displayed in (a)–(e) for SNP 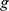 = 1, 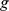 = 2,…, 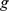 = 5, respectively, under the Bayesian unconditional likelihood model.

**Figure 2:**
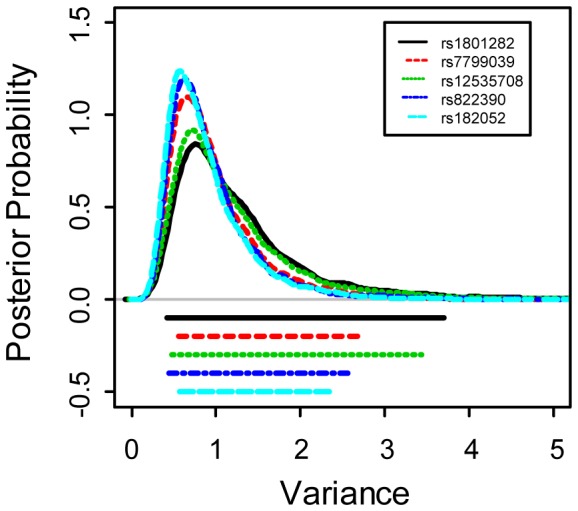
The posterior distributions of 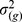 for 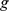 = 1, …, 5 SNP, respectively, under the Bayesian unconditional likelihood model.

To investigate the existence of the cross-level GE interaction, we list in Table 2 the posterior means and standard deviations of the 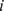-th category-specific genetic effects 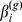 and variance 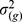 for each SNP *g*, and display the posterior distributions of the 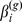 for SNPs *g* = 1, …, 5 in Figure 1(a)–(e). From Table 2, it is apparent that for rs1801282 (*g* = 1) the four category-specific means 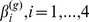, differ the most. This implies a relatively large variation of genetic effect across the 4 areas. The same pattern can be observed in the corresponding four posterior distributions of 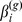 in Figure 1. The curves in Figure 1(a) are more scattered, as compared with the other four plots (Figure 1(b), (c), (d) and (e)), indicating substantially larger heterogeneity in the genetic effect of rs1801282 across the four categories. Another supporting evidence lies in the inference of 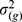 for *g* = 1, …, 5. The last column in Table 2 lists the posterior means of 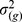 where the first SNP rs1801282 has the largest mean (1.31), implying again a larger heterogeneity. Figure 2 displays their corresponding distributions. Indeed, 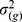 for rs1801282 is the most right skewed, representing an observable difference among areas. Further evidence manifests through the evaluation of the difference in risk across the four categories. For instance, for SNP *g* = 1, if the posterior probabilities of the risk, P(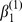
>0|*y*), P(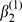
>0| *y*), P(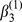
>0| *y*), and P(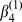
>0| *y*), deviate from each other, then this SNP has divergent effects among areas. Again, for rs1801282, the corresponding four posterior probabilities contain the largest degree of variation (Table 3).

**Table 2:** In the upper half of the table, numbers in each row are the posterior means and standard deviations of the area-specific genetic effects (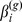) and variance (Var(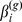) = 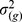) for each candidate SNP *g*.

	Posterior mean (se)					
SNP Covariates	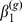 , I		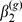 , II	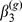 , III	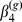 , IV	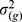
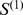 , rs1801282(*PPARG*_Pro12Ala)		1.01(0.83)	0.03(0.45)	−0.91(0.66)	−0.20(0.60)	1.31(0.94)
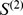 , rs7799039(*LEP*_G2548A)		0.25(0.71)	0.49(0.60)	0.65(0.65)	0.21(0.84)	1.03(0.68)
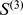 , rs12535708(*LEP*_H1328082)		9.90(5.11)	10.02(5.06)	10.71(5.09)	10.92(5.11)	1.22(0.84)
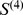 , rs822390(*APM1*_G-7950T)		0.52(0.55)	0.18(0.44)	0.64(0.57)	0.61(0.52)	0.97(0.64)
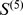 , rs182052(*APM1*_A-10066G)		−0.08(0.27)	0.27(0.20)	0.54(0.25)	0.12(0.26)	0.91(0.56)
SNP-SNP interaction						
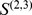 , rs7799039× rs12535708			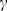	−10.70 (5.06)		
Other variance components						
Among areas, Var( 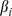 )			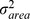	0.62(0.40)		
Among pairs, Var( 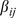 )			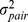	0.22(0.05)		

The bottom half of the table contains posterior means and standard deviations for parameters of the SNP-SNP interaction, and for variance component parameters.

**Table 3:** Numbers are 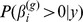, the posterior probability of 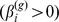, for the 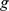-th SNP in the 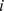-th category (area) under the unconditional model.

SNP, *S* ^(*g*)^	Category I	Category II	Category III	Category IV
rs1801282	0.90	0.53	0.07	0.37
rs7799039	0.63	0.80	0.84	0.60
rs12535708	1.00	1.00	1.00	1.00
rs822390	0.83	0.66	0.87	0.89
rs182052	0.38	0.91	0.99	0.67

These findings consistently imply the existence of the cross-level interaction between this SNP and the contextual variable. To be specific, the analyses suggest that, even among those residents with the same genotype C/G for rs1801282, living in an area with low availability of exercise facilities (category I) leads to a higher risk of being metabolically diseased. In other words, the G allele (also called the Ala allele in *PPARG*_Pro12Ala) is considered protective under categories II-IV, but not under category I. In fact, we have conducted Bayesian model selection using the deviance information criterion (DIC) to compare the model with GE interaction and without (assuming 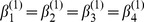). The former model containing the cross-level GE interaction term was selected.

This pattern of interaction, however, is not shown for other SNPs. For example, the second SNP (rs7799039) in Figure 1(b) and the fourth SNP (rs822390) in Figure 1(d) reveal only mild variation; while the third SNP (rs12535708) in Figure 1(c) and the fifth SNP (rs182052) in Figure 1(e) show almost no difference in the 95% credible intervals across the four categories. In addition, the density functions of 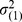, 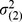, …, and 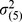 differ from each other, which implies different patterns in heterogeneity among the 5 SNPs.

### SNP-SNP Interaction

To evaluate the existence of interaction between SNPs, we examine the posterior distribution of 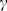 for the SNP-SNP interaction between rs7799039 and rs12535708. Notice that the posterior distribution of 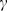 is left-skewed with a mean of −10.70 and a 95% credible interval (−21.8, −3.5), indicating a protective effect when these two SNPs simultaneously carry the G/G and A/A genotypes, respectively (Table 2 and Figure 1(f)). The overall aggregation of effects, however, shrinks toward zero when accounting for both single-SNP effects. For instance, in category I, the sum of the average odds ratios for genotype G/G for rs7799039, A/A for rs12535708, and their interaction is about exp(0.25+9.90–10.70) = exp(−0.55), resulting in an odds ratio of approximately 0.58( = exp(−0.55)). In other words, the interaction diminishes the two strong first-order genetic effects.

## Discussion

The research reported here was motivated by a matched case-control metabolic syndrome study where interest lay in the interaction between genes and contextual variables, as well as SNP-SNP interaction. Such cross-level GE interaction can be as important as the individual level Ge interaction, and thus its influence should not be overlooked. The statistical inference of GE, however, is not straightforward. We adopted here a Bayesian model with random effects to investigate the genetic and environmental interactions by examining the extent to which the allelic effects differ across various categories of the contextual variable. Though many studies have been proposed to test the interaction between genes and environment at the individual level, this Bayesian approach, to the best of our knowledge, is the first Bayesian analysis to infer the cross-level interaction between individual genetic and contextual residential area effects. The advantages of employing a Bayesian unconditional likelihood model are its flexibility in model construction for complex models; the straightforwardness in deriving the posterior distribution of the variance 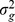 to assess cross-level interaction; and the ability to estimate multidimensional parameters simultaneously.

### Biological Implications

The results indicated that the effect of rs1801282 is likely to be modified by the contextual factor in the sense that individuals with the mutant genotype have higher risk, particularly when living in an area with low availability of exercise facilities. On the basis of the large posterior variability in genetic effects across areas, we conclude that this effect is modulated by the residential environmental factor. Similar observations have been previously reported in other studies where the rs1801282 SNP was shown to exacerbate the negative effect of poor individual exercise habits [24]–[27]. Others have observed that physical inactivity and sedentary lifestyle may interfere with optimized expression of the “thrifty” genes [28] and that the availability of recreational resources can relate directly to an individual’s physical activity level [39]. These findings are in agreement with our finding that the availability of recreational resources will interact with the effect of rs1801282 on metabolic syndrome in such a way that living in areas with lower exercise facility availability will increase the risk of disease, particularly for individuals carrying the mutant type of rs1801282.

The model we have introduced here for the matched design can accommodate SNP-SNP interaction as well. For instance, SNPs rs12535708 and rs7799039 locate closely in the gene *LEP* on chromosome 7q31, where rs7799039 is in the 5′ regulatory promoter region and rs12535708 is at the transcription-factor binding site. The strong LD between these two SNPs has been documented earlier [40], and the existence of their statistical interaction indicates that these two SNPs may be involved in similar functional pathways. An existing study reported that rs7799039 cannot add further information when other multimarker haplotypes containing rs12535708 have been included for analysis [40]. Our results replicate the finding that, when both SNPs are present in the model, rs12535708 exhibits strong association (the posterior modes of 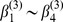 are all around 10), while the influence of rs7799039 is mild (posterior modes of 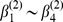 are between 0 and 1). When accounting for both markers along with their interaction, the estimated odds ratios of being metabolic, for persons carrying genotype G/G for rs7799039 and A/A for rs12535708, are 0.57, 0.83, 1.02, and 1.54 for the four categories, respectively. Each of the resulting values implies the existence of SNP-SNP interaction, and the heterogeneity among the four odds ratios provides evidence for GE interaction. Further research about the functional polymorphism of these two SNPs, or haplotype analysis, would be worth pursuing to unravel their biological interplay.

### Other Statistical Models for GE interaction

#### 1. Bayesian conditional logistic regression model

If only some of the previously mentioned parameters are of interest, then their inference can be made under the Bayesian conditional logistic regression (BCLR) model assuming 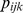 = 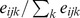, and a log link formulation on 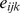


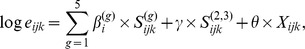
where for each SNP *g*, 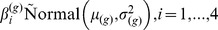. Table S2 displays the model and parameters for comparison. Table 4 lists the posterior means and standard errors of 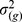 for 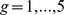. The posterior distributions of 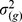 under the Bayesian conditional logistic regression do not vary much from that under the Bayesian unconditional likelihood model. In addition, we have investigated the pattern based on a limited simulation study with only 10 replications, each containing five SNPs for 100 cases and 100 matched controls in every one of the 4 areas. The consistency in the inference of variance between the Bayesian unconditional and conditional models stays clearly. However, as compared with the previous unconditional model, the disadvantages are that one is unable to infer the sampling variability among areas or within each pair under this BCLR, and the computational difficulty is greatly elevated under this setting if the number of genetic markers is large. Our experience shows that even when the computation under BCLR reaches convergence, the computation time for BCLR is about 4.5 times that of the Bayesian full likelihood model, when computed with an Intel core i7-2620M (2.7/3.4GHz) dual-core processor.

**Table 4:** The variance parameters represent the variability among areas for each SNP.

Corresponding SNPs	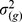
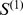 , rs1801282(*PPARG*_Pro12Ala)	1.34 (1.02)
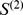 , rs7799039(*LEP*_G2548A)	1.03 (0.64)
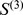 , rs12535708(*LEP*_H1328082)	1.28 (0.93)
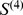 , rs822390(*APM1*_G-7950T)	1.00 (0.61)
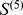 , rs182052(*APM1*_A-10066G)	0.93 (0.56)

Numbers are posterior means and standard deviations of variance components under the Bayesian conditional logistic regression model.

Although the BCLR model could be viewed as a workable alternative model in this case, its computational burden and lack of information on sampling variability within clusters hinder its use in such a cross-level GE study. The unconditional likelihood approach uses matched-pair specific random effects to account for the selection bias, while the conditional approach adopts matched-pair specific intercepts [41]. Another reason for favoring the Bayesian unconditional likelihood approach is that the full likelihood model can accommodate complex nested structures easily and intuitively, such as those involving matched cases and controls in the same category. These various sources of dependence can be modeled straightforwardly.

#### 2. Models with cross-product terms

Other alternative for handling the cross-level GE interaction in a matched design is through the use of cross-product terms of dummy variables for areas and SNP covariates. Some possibilities are outlined in the supporting information (Text S3).

We do not recommend however the use of these other models with cross-product terms, because the large number of coefficients of GE interaction often leads to failure in the MCMC computations. Other non-Bayesian multi-level models with the MQL option in MLwiN and glmmPQL in R are theoretically possible, but the computation for this matched case-control metabolic syndrome study collapses when it runs into a non-positive definite matrix during the iteration procedure.

### Further Notes

Some further notes are worth mentioning. First, for the sensitivity analysis of the posterior inference, we have tried other prior distributions. For example, we have used different gamma distributions for the precision parameters (the inverse of the variance components), and obtained similar conclusions. Second, we have adopted a Bayesian hierarchical model with no GE interaction for rs1801282, and performed model selection with DIC. The model with the cross-level GE was selected, and therefore the posterior probability under the GE model was evaluated for inference. Last but not least, although Bayesian models may at first seem complicated with respect to their formulation and computation, the advancement of MCMC methods has greatly enhanced the feasibility of using Bayesian inference in daily practice. Several software applications, such as WinBUGS, R, and MLWin, are handy for carrying out Bayesian analysis. The model presented here for analyzing interaction between genes and contextual variables could achieve broad applicability with the aid of these statistical analysis tools.

## Supporting Information

Table S1 **The formulation and parameter interpretation of the unconditional likelihood Bayesian model.**
(DOCX)

Table S2 **The formulation and parameter interpretation of the Bayesian conditional logistic regression model.**
(DOCX)

Text S1 **Complete specification of the Bayesian model.**
(DOC)

Text S2 **WinBUGS code of the Bayesian model.**
(DOCX)

Text S3 **Model formulation and parameter interpretations with cross-product terms for cross-level interaction.**
(DOCX)
