# Supplementary material for: Inference of Cross-Level Interaction between Genes and Contextual Factors in a Matched Case-Control Metabolic Syndrome Study: A Bayesian Approach
Source: PLoS One. 2013 Feb 20;8(2):e56693. doi: 10.1371/journal.pone.0056693 (PMC3577698; doi:10.1371/journal.pone.0056693)
Supplement: Table S1 — The formulation and parameter interpretation of the unconditional likelihood Bayesian model. (DOCX) [file pone.0056693.s001.docx]

Table S1. The formulation and parameter interpretation of the unconditional likelihood Bayesian model.

| **The unconditional likelihood Bayesian model** | |
| --- | --- |
|  | *Y_ijk_* \| *p_ijk_* ~ Bernoulli (*p_ijk_*)  where logit (*p_ijk_*)=$\sum_{g=1}^{5} \beta_{i}^{(g)}\times S_{ijk}^{(g)}+\gamma\times S_{ijk}^{(2,3)}+\theta\times X_{ijk}+\beta_{i}+\beta_{ij}$.  $\beta_{i}^{(g)}$, $\gamma$, $\theta$, $\beta_{i}$ and $\beta_{ij}$ follow Normal prior distributions  **Note:** $i$=1, …,4 with 1 for level I of very low exercise availability; $j$=1,…, 268 for the index of pairs; $k$=1, …, *n_j_* for the index of individuals in the $j$-th pair with *n_j_* standing for the number of individuals in the pair. |
| Parameter Interpretation:  Cross-level interaction (random effects): $\beta_{i}^{(g)}$ and Var($\beta_{i}^{(g)}$)=$\sigma_{\text{(}g\text{)}}^{2}$  SNP-SNP interaction (random effects): $\gamma$  Random effect for other covariates: $\theta$  Area-specific random effect: $\beta_{i}$  Pair-specific random effect: $\beta_{ij}$  Explanatory Variables:  $S_{ijk}^{(g)}$: coding of SNP $g$ for the $k$-th individual in the $j$-th pair of the $i$-th category  $S_{ijk}^{(2,3)}$: coding of interaction of SNPs 2 and 3  $X_{ijk}$: covariate variable, not used here  Response Variable: $Y_{ijk}$, disease status of the $k$-th individual in the $j$-th pair of the $i$-th category | |
