# Supplementary material for: Inference of Cross-Level Interaction between Genes and Contextual Factors in a Matched Case-Control Metabolic Syndrome Study: A Bayesian Approach
Source: PLoS One. 2013 Feb 20;8(2):e56693. doi: 10.1371/journal.pone.0056693 (PMC3577698; doi:10.1371/journal.pone.0056693)
Supplement: Table S2 — The formulation and parameter interpretation of the Bayesian conditional logistic regression model. (DOCX) [file pone.0056693.s002.docx]

Table S2. The formulation and parameter interpretation of the Bayesian conditional logistic regression model.

| **Bayesian conditional logistic regression model** | |
| --- | --- |
|  | *Y_ijk_* \| *p_ijk_* ~ Bernoulli (*p_ijk_*)  where $p_{ijk}$=$e_{ijk}/\sum_{k} e_{ijk}$  and   $\log e_{ijk}\text{=}\sum_{g=1}^{5} \beta_{i}^{(g)}\times S_{ijk}^{(g)}+\gamma\times S_{ijk}^{(2,3)}+\theta\times X_{ijk}$ $\beta_{i}^{(g)}$, $\gamma$, and $\theta$ follow Normal prior distributions |
| Parameter Interpretation:  Cross-level interaction (random effects): $\beta_{i}^{(g)}$ and Var($\beta_{i}^{(g)}$)=$\sigma_{\text{(}g\text{)}}^{2}$  SNP-SNP interaction (random effect): $\gamma$  Random effect for other covariates: $\theta$ | |
