# Supplementary material for: Inference of Cross-Level Interaction between Genes and Contextual Factors in a Matched Case-Control Metabolic Syndrome Study: A Bayesian Approach
Source: PLoS One. 2013 Feb 20;8(2):e56693. doi: 10.1371/journal.pone.0056693 (PMC3577698; doi:10.1371/journal.pone.0056693)
Supplement: Text S1 — Complete specification of the Bayesian model. (DOC) [file pone.0056693.s003.doc]

**Supporting Information: Complete specification of the Bayesian model**

The response variable $Y_{ijk}$ follows a Bernoulli distribution,

$Y_{ijk}$| ~ Bernoulli (),

where the probability of diseased $p_{ijk}$ is associated with the genetic components $S^{(1)},...,\text{ }S^{(5)}$ and a SNP-SNP interaction $S^{(2,3)}$ through the logit link function,

logit (*p_ijk_*)=$\sum_{g=1}^{5} \beta_{i}^{(g)}\times S_{ijk}^{(g)}+\gamma\times S_{ijk}^{(2,3)}+\theta\times X_{ijk}+\beta_{i}+\beta_{ij}$.

The prior distributions for the category-specific random coefficients $\beta_{i}^{(g)}$ are

$$\beta_{i}^{(g)}\sim\text{Normal}\left(\mu_{(g)}\text{,}\sigma_{(g)}^{2}\right),\text{ }i=1,...,4$$

for $g=1,...,5$ SNPs, where the hyper-parameters $\mu_{(g)}$ follows non-informative N(0, 100) and $\sigma_{(g)}^{2}$ follows Inverse Gamma with parameters (3,3). The priors for the rest are $\gamma\sim$Normal (0,100), $\beta_{i}\sim$Normal$(\text{0,}\sigma_{area}^{2})$, $\beta_{ij}\sim$Normal$\text{(0,}\sigma_{pair}^{2}\text{)}$, and $\sigma_{area}^{2},\sigma_{pair}^{2}\sim$Inverse Gamma (3,3). Note that $\beta_{i}$ is the area-specific random effect and its variance parameter can account for the sampling variability among areas. The $\beta_{ij}$ is for pair-specific random effect and its variance component $\sigma_{pair}^{2}$ indicates the variation among pairs. The posterior inference of $\sigma_{pair}^{2}$ provides evidence of heterogeneity from pair to pair. In the study of metabolic syndrome, the $\theta$ is assumed a priori from Normal (0,100). These reference priors are chosen to represent vague information about the parameters.
