# Supplementary material for: Inference of Cross-Level Interaction between Genes and Contextual Factors in a Matched Case-Control Metabolic Syndrome Study: A Bayesian Approach
Source: PLoS One. 2013 Feb 20;8(2):e56693. doi: 10.1371/journal.pone.0056693 (PMC3577698; doi:10.1371/journal.pone.0056693)
Supplement: Text S2 — WinBUGS code of the Bayesian model. (DOCX) [file pone.0056693.s004.docx]

**Supporting Information: WinBUGS code for the Bayesian model**

model

{

for (i in 1:A){

#A: number of areas

beta.I[i]~dnorm(0.0,tau.I)

for (j in 1:(class[i+1]-class[i] )){

#class: cumulative frequency of number of matched pairs in each area

beta.J[i,j]~dnorm(0.0,tau.J)

for (k in 1:k.no[(class[i]+j)]){

#k.no: number of subjects in each matched pair

y[i,j,k] <- yy[(person[(class[i]+j)])+k]

#person: cumulative frequency of k.no

y[i,j,k] ~ dbern(p[i,j,k])

logit(p[i,j,k])<-beta.pparg[i] *pparg[(person[(class[i]+j)])+k]

+beta.Lep48[i] *Lep48[(person[(class[i]+j)])+k]

+beta.Lep82[i] *Lep82[(person[(class[i]+j)])+k]

+beta.Apm50[i] *Apm50[(person[(class[i]+j)])+k]

+beta.Apm66[i] *Apm66[(person[(class[i]+j)])+k]

+beta.gg*Lep48[(person[(class[i]+j)])+k]*Lep82[(person[(class[i]+j)])+k]

+beta.I[i]+beta.J[i,j]

}

}

}

beta.gg~ dnorm (0.0,0.001)

for (i in 1:A) {

beta.pparg[i]~ dnorm(u1, tau.b1)

beta.Lep48[i]~ dnorm(u2, tau.b2)

beta.Lep82[i]~ dnorm(u3, tau.b3)

beta.Apm50[i]~ dnorm(u4, tau.b4)

beta.Apm66[i]~ dnorm(u5, tau.b5)

}

u1~ dnorm (0.0,0.001)

u2~ dnorm (0.0,0.001)

u3~ dnorm (0.0,0.001)

u4~ dnorm (0.0,0.001)

u5~ dnorm (0.0,0.001)

tau.b1~ dgamma(3,3)

var.b1<-1/tau.b1

tau.b2~ dgamma(3,3)

var.b2<-1/tau.b2

tau.b3~ dgamma(3,3)

var.b3<-1/tau.b3

tau.b4~ dgamma(3,3)

var.b4<-1/tau.b4

tau.b5~ dgamma(3,3)

var.b5<-1/tau.b5

tau.I~ dgamma(3,3)

var.I<-1/tau.I

tau.J~ dgamma(3,3)

var.J<-1/tau.J

}
