# Supplementary material for: Inference of Cross-Level Interaction between Genes and Contextual Factors in a Matched Case-Control Metabolic Syndrome Study: A Bayesian Approach
Source: PLoS One. 2013 Feb 20;8(2):e56693. doi: 10.1371/journal.pone.0056693 (PMC3577698; doi:10.1371/journal.pone.0056693)
Supplement: Text S3 — Model formulation and parameter interpretations with cross-product terms for cross-level interaction. (DOCX) [file pone.0056693.s005.docx]

**Supporting Information Text S3:**

**Models with Cross-product Terms for Cross-level Interactions**

If the number of SNPs considered in the study is small, then one can test this interaction by testing the existence of the cross-product terms of the genetic and environmental variables. For instance, for the four categories of exercise facility availability (with low availability as the reference), three dummy variables $D_{1},\text{ }D_{2}$ and $D_{3}$ can be adopted. Then, for each of the 5 candidate SNPs in the metabolic syndrome association study, there are 3 cross-product terms for GE interaction. The relation between the probability $p_{ijk}$ and the covariates can then be written in the logit scale as

$$\begin{matrix} \text{logit}(p_{ijk})=\alpha_{ij}+\sum_{g=1}^{5} \beta^{(g)}\times S_{ijk}^{(g)} \\ \text{ }+\sum_{g=1}^{5} [\beta^{(D_{1},S^{(g)})}\times D_{1}\times S_{ijk}^{(g)}+\beta^{(D_{2},S^{(g)})}\times D_{2}\times S_{ijk}^{(g)}+\beta^{(D_{3},S^{(g)})}\times D_{3}\times S_{ijk}^{(g)}] \\ \text{ }+\gamma\times S_{ijk}^{(2,3)}+\theta\times X_{ijk} \end{matrix}$$

where $\alpha_{ij}$ is the pair-specific effect with the index $ij$. These $\beta^{(D_{l},S^{(g)})}$‘s act in a similar role to the $\beta$’s in the Bayesian full likelihood model discussed earlier. Note that the regression coefficients $\beta$’s are not indexed with $i,j$ or $k$ because they are assumed the same for all individuals. This model is listed in Table 1 of this file. The likelihood ratio test (LRT) for GE interaction implies no statistical significance for its existence (all p-values in the last column in Table 2 in this file are greater than 0.05). Even for the first SNP rs180128, the LRT for GE results in a mild p-value (p=0.07). The LRT values in the following table provide no information about how similar the genetic effects are across the four contextual levels.

Such analysis is feasible under the Bayesian approach. For example, if any probabilistic evaluation of coefficient of GE interaction is of interest, such as whether$\beta^{(D_{l},S^{(g)})}>0$, and if the Bayesian conditional approach is preferred, then letting $p_{ijk}$=$e_{ijk}/\sum_{k} e_{ijk}$, the link function becomes

$$\begin{matrix} \text{log}(e_{ijk})=\sum_{g=1}^{5} \beta^{(g)}\times S_{ijk}^{(g)} \\ \text{ }+\sum_{g=1}^{5} [\beta^{(D_{1},S^{(g)})}\times D_{1}\times S_{ijk}^{(g)}+\beta^{(D_{2},S^{(g)})}\times D_{2}\times S_{ijk}^{(g)}+\beta^{(D_{3},S^{(g)})}\times D_{3}\times S_{ijk}^{(g)}] \\ \text{ }+\gamma\times S_{ijk}^{(2,3)}+\theta\times X_{ijk} \end{matrix}$$

with $\beta^{(D_{l},S^{(g)})}\text{\textasciitilde Normal}\left(0\text{,}\sigma_{(g)}^{2}\right),\text{ }l=1,2,3$.

Table 1: Formulations and interpretations of two models with cross-product terms.

| **Non-Bayesian conditional likelihood model with cross-product terms** | | |
| --- | --- | --- |
|  | *Y_ijk_* | *p_ijk_* ~ Bernoulli (*p_ijk_*)  where $\begin{matrix} \text{logit}(p_{ijk})=\alpha_{ij}+\sum_{g=1}^{5} \beta^{(g)}\times S_{ijk}^{(g)} \\ \text{ }+\sum_{g=1}^{5} [\beta^{(D_{1},S^{(g)})}\times D_{1}\times S_{ijk}^{(g)}+\beta^{(D_{2},S^{(g)})}\times D_{2}\times S_{ijk}^{(g)}+\beta^{(D_{3},S^{(g)})}\times D_{3}\times S_{ijk}^{(g)}] \\ \text{ }+\gamma\times S_{ijk}^{(2,3)}+\theta\times X_{ijk} \end{matrix}$ | |
| Parameter Interpretation:  Cross-level interaction (fixed effects): $\beta^{(D1,S^{(g)})},\beta^{(D2,S^{(g)})},\beta^{(D3,S^{(g)})}$ for each SNP *g*  SNP-SNP interaction (fixed effects): $\gamma$ Fixed effect for other covariate: $\theta$ Pair-specific fixed effect: $\alpha_{ij}$ SNP specific fixed effect: *β*(*^g^*) | | |
|  | |  |
| **Bayesian conditional likelihood logistic regression model with cross-product terms** | | |
|  | *Y_ijk_* | *p_ijk_* ~ Bernoulli (*p_ijk_*)  where $p_{ijk}$=$e_{ijk}/\sum_{k} e_{ijk}$ and $\begin{matrix} \text{log}(e_{ijk})=\sum_{g=1}^{5} \beta^{(g)}\times S_{ijk}^{(g)} \\ \text{ }+\sum_{g=1}^{5} [\beta^{(D_{1},S^{(g)})}\times D_{1}\times S_{ijk}^{(g)}+\beta^{(D_{2},S^{(g)})}\times D_{2}\times S_{ijk}^{(g)}+\beta^{(D_{3},S^{(g)})}\times D_{3}\times S_{ijk}^{(g)}] \\ \text{ }+\gamma\times S_{ijk}^{(2,3)}+\theta\times X_{ijk} \end{matrix}$   *β*(*^g^*) , $\beta^{(D1,S^{(g)})},\beta^{(D2,S^{(g)})},\beta^{(D3,S^{(g)})}$, $\gamma$, and $\theta$ follow Normal prior distributions | |
| Parameter Interpretation:  Cross-level interaction (fixed effects): $\beta^{(D1,S^{(g)})},\beta^{(D2,S^{(g)})},\beta^{(D3,S^{(g)})}$ for each SNP *g*  SNP-SNP interaction (fixed effects): $\gamma$ Fixed effect for other covariate: $\theta$ SNP specific fixed effect: *β*(*^g^*) | | |

Table 2. Numbers are estimates and standard errors (se) of regression coefficients under the conditional logistic regression model. LRT tests the existence of GE interaction.

| Variables | estimate (se) | p-value | LRT $\chi^{2}$ (p value) |
| --- | --- | --- | --- |
| SNP |  |  |  |
| rs1801282 | 1.61 (1.12) | 0.15 |  |
| rs7799039 | 0.23 (0.83) | 0.79 |  |
| rs12535708 | 16.04 (997) | 0.99 |  |
| rs822390 | 0.34 (0.61) | 0.58 |  |
| rs182052 | -0.21 (0.35) | 0.54 |  |
| GE Interaction term |  |  |  |
| rs1801282×$D_{1}$ | -1.26 (1.22) | 0.30 |  |
| rs1801282×$D_{2}$ | -3.06 (1.40) | 0.03 |  |
| rs1801282×$D_{3}$ | -2.09 (1.34) | 0.12 |  |
|  |  |  | $\chi^{2}$=7.05 (0.07) |
| rs7799039×$D_{1}$ | 0.61 (1.12) | 0.59 |  |
| rs7799039×$D_{2}$ | 0.05 (1.22) | 0.97 |  |
| rs7799039×$D_{3}$ | -2.11 (2.05) | 0.30 |  |
|  |  |  | $\chi^{2}$=2.15 (0.54) |
| rs12535708×$D_{1}$ | 0.37 (1.78) | 0.84 |  |
| rs12535708×$D_{2}$ | 2.21 (2.06) | 0.28 |  |
| rs12535708×$D_{3}$ | 4.64 (2.69) | 0.08 |  |
|  |  |  | $\chi^{2}$=5.04 (0.17) |
| rs822390×$D_{1}$ | -0.36 (0.83) | 0.66 |  |
| rs822390×$D_{2}$ | 0.86 (0.97) | 0.38 |  |
| rs822390×$D_{3}$ | 1.56 (1.27) | 0.22 |  |
|  |  |  | $\chi^{2}$=3.83 (0.28) |
| rs182052×$D_{1}$ | 0.64 (0.41) | 0.12 |  |
| rs182052×$D_{2}$ | 0.98 (0.45) | 0.03 |  |
| rs182052×$D_{3}$ | 0.48 (0.47) | 0.31 |  |
|  |  |  | $\chi^{2}$=5.16 (0.16) |
| GG interaction |  |  |  |
| rs7799039×rs12535708 | -17.10 (997) | 0.99 |  |
